# Topical Application of RNAi Therapy Using Surface-Modified Liposomes for Treating Retinal-Vein Occlusion

**DOI:** 10.3390/molecules30122622

**Published:** 2025-06-17

**Authors:** Taishi Shiratori, Takaaki Ito, Anri Nishinaka, Ryosuke Matsumiya, Eriko Yamazoe, Hirofumi Takeuchi, Hideaki Hara, Kohei Tahara

**Affiliations:** 1 Laboratory of Pharmaceutical Engineering, Gifu Pharmaceutical University, 1-25-4 Daigaku-Nishi, Gifu 501-1196, Japanito-ta@gifu-pu.ac.jp (T.I.); lucky.ryosuke.0414@gmail.com (R.M.); yamazoe-e@gifu-pu.ac.jp (E.Y.); takeuchi@gifu-pu.ac.jp (H.T.); 2 Laboratory of Molecular Pharmacology, Gifu Pharmaceutical University, 1-25-4 Daigaku-Nishi, Gifu 501-1196, Japanhidehara@gifu-pu.ac.jp (H.H.); 3 Laboratory of Advanced Pharmaceutical Process Engineering, Gifu Pharmaceutical University, 1-25-4 Daigaku-Nishi, Gifu 501-1196, Japan

**Keywords:** retina, eye drop, drug delivery, thermoresponsive gel, retinal-vein occlusion, nucleic acid

## Abstract

Retinal diseases can result in blindness and visual impairment. They represent a significant medical burden and adversely affect life expectancy. Recently, antibody- and nucleic acid-based pharmaceuticals have increasingly been used to treat retinal diseases, with improvement or cure as the goal; however, these drugs are currently only administered by intravitreal injection. In this study, we present a novel approach to treating retinal diseases using eye drops that contain PnkRNA, a single-stranded RNA nucleic acid. PnkRNA-loaded liposomes were shown to effectively deliver retinal drugs and significantly inhibit retinal thickening in a mouse retinal-vein occlusion model. Cationic modification of the liposome surface enhanced the delivery of nucleic acids and therapeutic efficacy. Moreover, to reduce the frequency of eye-drop administration, liposomes were incorporated into the thermoresponsive gels. This formulation provided sustained retinal delivery and exhibited superior therapeutic efficacy compared with liposomal eye drops. This nucleic acid retinal delivery technology represents a significant advancement in drug-delivery technology, offering a safe and simple treatment for retinal diseases.

## 1. Introduction

Posterior ocular diseases, particularly retinal diseases, can cause severe visual impairment and blindness because of the important role of the posterior eye in maintaining visual acuity and the visual field. The majority of retinal diseases progress slowly and initially present with few symptoms. This complicates treatment and results in poor compliance and an increased incidence of visual impairment. Greater than 80% of global blindness cases occur in individuals over 50 years old; thus, retinal diseases are poised to become a significant problem in developed countries with aging populations [1].

Retinal-vein occlusion (RVO) can lead to retinal necrosis, often resulting in irreversible visual impairment and blindness [2]. It is primarily caused by high blood pressure and atherosclerosis [3], which compromise retinal venous circulation. This leads to ischemia, which reduces the supply of nutrients and oxygen to the retina and ultimately triggers progressive retinal tissue degeneration. Ischemic retinal tissue releases vascular endothelial growth factor (VEGF), which induces the expansion of the area of venous occlusion and worsens the symptoms. Current treatments for RVO include surgery, such as laser photocoagulation, and VEGF-targeted antibody therapy.

The most common route of administration for VEGF-targeted antibodies for treating RVO is direct injection into the vitreous body. This approach can increase the local drug concentration and therapeutic efficacy; however, it is an invasive procedure that is difficult for patients to self-administer. It is also associated with adverse effects, including intraocular inflammation, cataracts, and, in rare cases, retinal detachment [4]. Moreover, the frequent administration of vitreous drugs is required at intervals of 2 and 6 weeks. The invasiveness and time constraints associated with hospital visits also significantly reduce patient compliance. Eye drops are a noninvasive and simple drug-delivery alternative; however, the high molecular weight of VEGF-targeted antibodies and the presence of drug penetration inhibitory mechanisms [5,6], such as tear fluid clearance and ocular surface protection, limit the feasibility of retinal delivery using eye drops. Therefore, it is necessary to develop a drug-delivery system (DDS) that achieves efficient posterior ocular delivery.

For topical drug delivery via eye-drop administration, nanocarrier-based DDSs represent a promising ophthalmic drug delivery system [7,8]. Nanocarriers, such as liposomes composed of phospholipids, can stabilize a drug, enhance tissue delivery, and promote its sustained release [9]. Nagai et al. reported that the particle characteristics of nanocarriers significantly influence pharmacokinetics, with particle size affecting drug retention on the ocular surface and excretion through the tear ducts [10]. For example, particles <100 nm exhibit high corneal permeability, but excessively small particles may be rapidly cleared by the lacrimal glands. We previously demonstrated that reducing liposomes to submicron sizes enabled the eye-drop delivery of diclofenac to the retina [11,12]. Furthermore, liposomes modified with poly-L-lysine, a cationic low-molecular-weight polymer, prolonged ocular surface residence time and enhanced retinal delivery [13]. These results suggest that even macromolecular drugs can be delivered to the posterior segment using eye drops if they are encapsulated in nanocarriers.

The current study aimed to evaluate the potential of nucleic acid drugs as a therapeutic modality for RVO. Nucleic acid drugs, such as pegaptanib (Macugen^®^; Eyetech Pharmaceuticals Inc., New York, NY, USA), have been used for the treatment of age-related macular degeneration, a type of retinal disease. Nucleic acid drugs are promising candidates for delivery using nanocarriers because of their smaller molecular size and versatility resulting from nucleic acid synthesis technology compared with antibody drugs. Preclinical and clinical studies are currently evaluating nucleic acid therapies for retinal diseases, with a primary focus on inherited retinal disorders [14]. Although some studies have examined polymer-based delivery systems [15], adeno-associated virus (AAV) vectors are considered the gold standard for ophthalmic drug delivery [14]; however, most AAV-based therapies are administered via intravitreal injection. Despite limited studies on nucleic acid eye drops, Nishida et al. reported that siRNA-loaded liposomal eye drops may be effective for retinal delivery [16]. This suggests that the posterior segment delivery of nucleic acids is a feasible strategy; however, no studies to date have provided evidence of therapeutic efficacy.

The present study is divided into two sections: (1) evidence supporting the therapeutic efficacy of nucleic acid-loaded liposomes for RVO, and (2) efforts to enhance the formulation to reduce the frequency of eye-drop administration. PnkRNA was used as a nucleic acid loaded into liposomes as a drug carrier [17]. PnkRNA degrades mRNA through RNA interference and can target specific genes. In this study, PnkRNA was designed to target VEGF, which provides a mechanism of action distinct from that of pegaptanib, an aptamer-based VEGF inhibitor. As a single-stranded RNA, PnkRNA exhibits enhanced in vivo stability and can suppress the induction of nonspecific inflammatory responses via Toll-like receptors, which is a problem for double-stranded nucleic acid drugs, such as siRNA [18]. PnkRNA was developed as a treatment using transforming growth factor-β1 (TGF-β1), targeting idiopathic pulmonary fibrosis, coronavirus disease 2019 (COVID-19), and corneal neovascularization [19,20]. To increase cell permeability, cationic modifiers, such as stearoyl-octa-arginine (R8) or stearylamine (SA), are used for the modification of PnkRNA-loaded liposomes. Liposomes modified with R8 or SA exhibit lower ocular irritation compared with other cationic surface modifiers. They have been shown to enhance nucleic acid uptake by cells [21,22]. In the present study, PnkRNA-loaded liposomes were administered to mouse eyes, and the therapeutic efficacy of the PnkRNA-loaded liposome eye drops was demonstrated by observing the progression of retinal edema following administration.

Second, although eye-drop administration may be convenient, the high frequency of administration required may prove stressful for patients. The majority of the administered ophthalmic solution is rapidly diluted by mixing with tear fluid and draining into the tear ducts and systemic circulation, with an estimated clearance rate of 15–30% per minute [7]. The efficacy of ophthalmic therapy depends on good medication compliance, and the frequency of eye-drop administration is a critical determinant [23]. Accordingly, the second part of the present study focused on thermoresponsive gels that increase the viscosity of eye drops and their residence time on the ocular surface, to reduce the frequency of administration. Thermoresponsive gels consisting of methylcellulose (MC) exist in a solid state below the lower critical solution temperature. Upon warming, the water molecules dehydrate from the MC to induce aggregation, which results in a transition to a gel state [24]. Thermoresponsive gels exist in a low-viscosity solution before administration, thereby reducing the pressure during application, and after administration. They gel in response to the ocular surface temperature, thereby improving drug retention on the surface. Our overall goal was to design a formulation with liposomes added to thermoresponsive gels to reduce the frequency of liposomal eye-drop administration, thereby facilitating a practical treatment regimen for RVO.

## 2. Results and Discussion

### 2.1. PnkRNA-Loaded Liposomal Eye Drops Inhibit Retinal Edema in Retinal-Vein Occlusion Model Mice

#### 2.1.1. Characterization of PnkRNA-Loaded Liposomes

Because the action of PnkRNA occurs intracellularly, it is necessary to introduce the molecule intracellularly for it to be effective; however, PnkRNA is a negatively charged, water-soluble macromolecule derived from phosphate groups, thus its transfection efficiency into the cytoplasm is limited. We previously achieved corneal delivery of PnkRNA through eye-drop administration of PnkRNA-loaded liposomes [20]. The results indicated that cationic modification of the liposomal surface charge enhanced drug delivery to the retina [13]. Nagai et al. previously showed that the permeability of nanoparticles administered through eye drops is influenced by their particle size [10]. Therefore, in the present study, PnkRNA was loaded into cationic liposomes with a particle size range of 100–200 nm. The composition and particle properties of the liposomes prepared in this study are listed in Table 1 and Table 2.

Unmodified liposomes (Unmodified-Lip), composed solely of lipid and cholesterol, exhibited a neutral to negative surface charge. In contrast, liposomes modified with R8 (R8-Lip) had a positive charge of 43.4 mV, indicating that it was successfully attached to the liposomal surface. Modification with R8 increased the particle size from 140.7 nm to 183.9 nm and the polydispersity from 0.062 to 0.167, while improving the PnkRNA loading efficiency from 18.8% to 99.7%. Cationic modification of liposomes increases particle size because of hydrogen bonding and electrostatic interactions with negatively charged substances in the liposome suspension [13]. These interactions may promote liposomal adhesion and aggregation. The observed increase in particle size and polydispersity, as well as the improvement in loading efficiency, may be attributed to the formation of electrostatic interactions between R8 and PnkRNA. During liposome formation via extrusion from multilamellar vesicles, the PnkRNA present in the aqueous phase, either within the inner aqueous compartment or adsorbed onto the vesicle surface, may have been effectively loaded into the liposomes through electrostatic interactions with R8.

#### 2.1.2. Mouse Retinal Delivery of PnkRNA by Eye-Drop Administration

**Figure 1:**
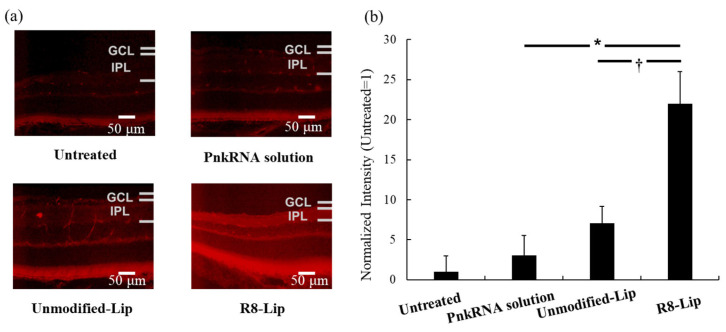
Mouse retinal delivery of PnkRNA through eye-drop administration. GCL, ganglion cell layer; IPL, inner plexiform layer; Lip, liposome; and R8-Lip, stearoyl-octa-arginine (R8) modified liposome. (**a**) Epifluorescence microscopic images of the mouse retina 30 min after TAMRA-labeled PnkRNA eye-drop administration. (**b**) Fluorescence intensity in the mouse IPL after 30 min of eye-drop administration (n = 6, mean ± standard error of the mean [SEM]). * *p* < 0.05 vs. PnkRNA solution, and † *p* < 0.05 vs. Unmodified-Lip (one-way analysis of variance (ANOVA) followed by Tukey’s multiple comparison test).

Considering the previous finding that the retinal delivery of low-molecular-weight drug eye drops reaches a maximum 30 min post-administration [11], the retinal delivery of PnkRNA in mice was evaluated 30 min following the first eye drop of TAMRA-labeled PnkRNA (Figure 1a). To enhance the visibility of retinal tissue in the Control group, the exposure time was intentionally extended to enable the detection of background autofluorescence. The TAMRA-labeled PnkRNA-loaded R8-modified liposomes (R8-Lip) exhibited stronger TAMRA-derived fluorescence compared with the other eye drops. Figure 1b shows the intensity of the TAMRA fluorescence at the IPL. The IPL is an appropriate target for evaluating retinal deliverability because of its proximity to the ganglion cell layer (GCL), which contains retinal ganglion cells. The death of retinal ganglion cells is common in several ophthalmic disorders, including optical neuropathy and retinal-vein occlusive disease [11]. The TAMRA-labeled PnkRNA-loaded unmodified liposomes (Unmodified-Lip) enhanced PnkRNA retinal delivery compared with that of the PnkRNA solution. Moreover, R8-Lip significantly enhanced PnkRNA retinal delivery relative to that of the PnkRNA solution and Unmodified-Lip. The cationic surface of R8 on the liposome may induce the long-term retention of liposomes on the ocular surface, thereby facilitating their uptake into the ocular tissue [13]. Of note, although the same quantity of PnkRNA (0.18 nmol/18 µL/mouse) was administered to the mouse eye in all eye drops, the loading efficiencies of the Unmodified-Lip and R8-Lip were markedly different (Table 2; 18.8% and 99.9%, respectively). It is unclear whether the mechanism through which R8 modification improves the retinal delivery of PnkRNA results from electrostatic interaction with ocular tissues or increased loading efficiency. Nevertheless, these findings emphasize the significance of loading PnkRNA into liposomes and modifying them with R8 to enhance their delivery to the mouse retina.

#### 2.1.3. Retinal Edema Therapeutic Evaluation of a Retinal-Vein Occlusion Mouse Model Following PnkRNA-Loaded Liposome Eye-Drop Administration

**Figure 2:**
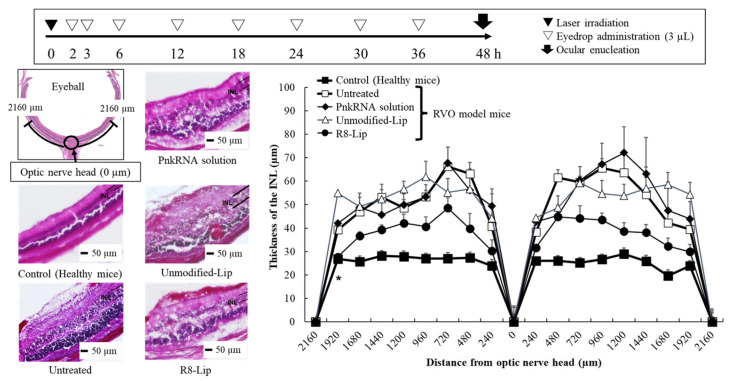
Thickness of the inner nuclear layer (INL) following definite intervals of eye-drop administration in retinal-vein occlusion (RVO) model mice. Lip, liposome; R8-Lip, stearoyl-octa-arginine (R8)-modified liposome. Each value represents the mean ± standard error of the mean (SEM) of eight measurements. * *p* < 0.05 vs. untreated (one-way ANOVA followed by Tukey’s multiple comparison test).

One of the clinical symptoms associated with RVO is retinal edema. PnkRNA eye drops were administered to RVO model mice, and their effect was evaluated by measuring retinal thickness in mouse ocular tissue sections. In RVO model mice, retinal edema occurs in the INL, which comprises glial and amacrine cells [25]. Figure 2 shows the evaluation scheme, retinal images, and INL thickness at 240 μm intervals centered on the optic nerve. The INL thickness was 12–40 µm thicker in the RVO model mice (untreated group) compared with that in the healthy mice (control group), indicating the presence of laser irradiation-induced retinal edema. The PnkRNA solution and Unmodified-Lip did not inhibit retinal edema progression. In contrast, R8-Lip-treated RVO model mice exhibited a significant reduction in INL thickening compared with the untreated group. These results are consistent with previous findings of improved retinal delivery (Figure 1), thereby supporting the therapeutic efficiency of PnkRNA administered via eye drops. Of note, PnkRNA eye drops administered after laser irradiation resulted in a therapeutic effect, suggesting that R8-Lip may also be effective even after the development of RVO.

The results suggest that nucleic acids can penetrate ocular defense mechanisms; however, we did not determine the retinal delivery pathway of PnkRNA, thus further studies are required. In a preliminary experiment, VEGF-mRNA levels in RVO model mice following eye-drop administration of PnkRNA-loaded liposomes were lower compared with those in the untreated group, although the difference was not statistically significant (Figure S3). Future studies should incorporate not only tissue section imaging and mRNA expression analyses, but also protein determination, including cytokine quantification. However, the accurate measurement of cytokine levels in mouse retinas remains technically challenging because of the limited tissue volume available for analysis. Yi et al. reported the retinal delivery of approximately 2000 Da peptide eye drops and their effects on age-related macular degeneration [26]. It is noteworthy that the modification of the peptide with hexa-arginine, an oligoarginine similar to R8, enabled retinal delivery and exhibited efficacy. The retinal delivery of liposomes by eye-drop administration may occur through three distinct pathways: the systemic, corneal, and noncorneal pathways [27]. Retinal delivery via the systemic route is ineffective, as the retinal amount detected on the side of the eye opposite to where the drops were administered was previously considered negligible [28]. Corneal tissue consists of three distinct layers, including the epithelium, stroma, and endothelium. The epithelium and endothelium layers are hydrophobic and form tight junctions, whereas the stroma is hydrophilic and strongly inhibits drug penetration into the cornea. As previously shown, liposomal eye drops are predominantly localized to the epithelium, suggesting that the corneal pathway is not a major delivery route [11]. In retinal flat-mount images following treatment with C6-loaded liposome eye drops, a concentration gradient of C6 was observed from the iris and ciliary body to the optic nerve head [28]. These findings indicate that the retinal delivery of liposomal eye drops primarily occurs through noncorneal pathways; however, the delivery mechanism is likely diverse and complex, involving various factors, such as the molecular weight of the drug, the particle properties of the carrier, and the substrate-specific delivery pathways. Moreover, it is important to consider liposomal retinal delivery not only from a pharmaceutical standpoint, but also in the context of the underlying pathology. The administration of C6-loaded liposomes via eye drops to the RVO model mice resulted in a significant increase in retinal delivery compared with that in healthy mice (Figure S4). This enhancement may be attributed to the RVO-mediated impairment of ocular-barrier function. Therefore, a comprehensive and systematic analysis is needed to fully elucidate the underlying mechanisms.

### 2.2. Liposome-Loaded Thermoresponsive Gels Reduce the Number of PnkRNA Eye Drop Treatments

#### 2.2.1. Characterization of the Liposome-Loaded Thermoresponsive Gels

The results presented in Section 2.1 indicate that PnkRNA-loaded liposomal eye drops show promise as an effective therapy for RVO; however, PnkRNA eye drops require frequent administration (four times daily), which may result in low patient compliance. Therefore, we attempted to reduce the frequency of administration of these eye drops by incorporating the liposomes into a thermoresponsive gel. Although the sol-gel phase transition temperature of the MC solution is 50–55 °C, the addition of SC and PEG4000 can reduce the sol-gel phase transition temperature to a value approaching that of body temperature. For example, Rysmon^®^ TG (Kissei Pharmaceutical Co., Ltd., Nagano, Japan), a glaucoma eye drop, forms a thermoresponsive gel with MC, SC, and PEG4000 [29]. Moreover, Itoh et al. demonstrated the effect of SR, a natural sugar alcohol, on an MC thermoresponsive gel [30]. Although there are several reports on the addition of microparticles to thermoresponsive gels [31], few have examined the particle properties of liposomal suspensions added to thermoresponsive gels. In the present study, we also evaluated the particle properties of the liposomes following their addition to a thermoresponsive gel.

The composition and particle properties of the liposomal eye drops and liposome-loaded thermoresponsive gels are listed in Table 3 and Table 4. The detailed preparation scheme is shown in Figure S2. Because of the cost of preparation, C6 was selected as the model drug, and SA was chosen as the cationic surface modifier. After a comprehensive examination of the thermoresponsive gel compositions (Table S1), two types of thermoresponsive gels were selected for study. These gels have the lowest possible viscosity before the administration of the eye drop (at room temperature, 10–25 °C) and an increase in viscosity at the ocular surface temperature (35 °C) following administration.

C6-loaded liposomes modified with SA (SA-Lip) had a particle size and polydispersity comparable with those of R8-Lip, as detailed in Section 2.1.1, with a slightly higher surface charge. The higher surface charge may be attributed to the absence of nucleic acids, which can form electrostatic interactions with SA. The surface properties of SA-Lip were altered in the thermoresponsive gels. The SA-Lip-loaded SR-TG and the SA-Lip-loaded SC-TG exhibited an increase in particle size and polydispersity and a negative shift in surface charge. In thermoresponsive gels consisting of nonionic MC, the SA-Lip may undergo an increase in the particle size and a shift in the surface charge toward neutrality because of the formation of an MC layer on its surface. Of note, these values result from the dilution of the thermoresponsive gels and may not accurately reflect the surface properties of the liposomes in the thermoresponsive gels; however, the observation of liposomes in the thermoresponsive gels yielded intriguing results (Section 2.2.2).

#### 2.2.2. Mouse Retinal Delivery of Coumarin 6 by Thermoresponsive Gel Eye-Drop Administration

**Figure 3:**
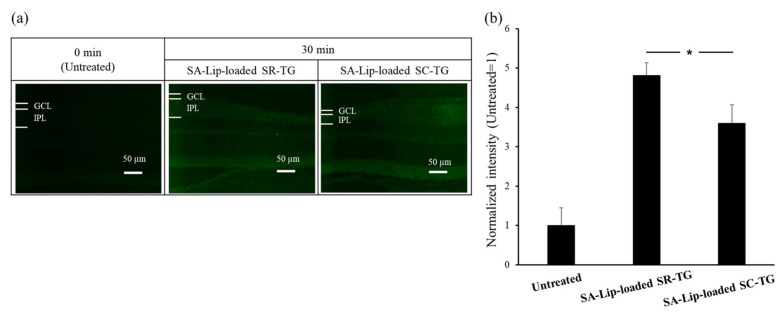
Mouse retinal delivery of coumarin 6 (C6) by single eye-drop administration of stearylamine-modified liposome (SA-Lip)-loaded thermoresponsive gels. GCL, ganglion cell layer; IPL; inner plexiform layer; SC, sodium citrate; SR, sorbitol; and TG, thermoresponsive gel. (**a**) Epifluorescence microscopic images of the mouse retina following the administration of eye-drop-loaded thermoresponsive gels. Scale bar: 50 μm. (**b**) Fluorescence intensity in the mouse IPL after eye-drop administration of the SA-Lip-loaded thermoresponsive gels (mean ± standard error of the mean [SEM], n = 6), * *p* < 0.05 vs. SA-Lip-loaded SC-TG (Aspin−Welch’s *t* test).

The objective of this study was to evaluate the drug-delivery efficiency of SA-Lip-loaded thermoresponsive gels on the retina. C6 fluorescence was detected in the retinal tissue. The ocular surface temperatures of the mice were measured at 36.2 °C ± 0.69 °C [mean ± SE (SEM), n = 4]. Figure 3 shows the retinal images and quantitative data for C6 fluorescence intensity at the IPL following the administration of the first eye drop after a 30 min interval. As described in Section 2.1.2 (Figure 1a), to enhance the visibility of the retinal tissue in the control group, the exposure time was extended to allow for the detection of background autofluorescence. C6 fluorescence was observed in the mouse retina for both the thermoresponsive gels, SR-TG and SC-TG. The relative fluorescence intensity of C6 (3.5- to 4.8-fold) was inferior to that of TAMRA-labeled PnkRNA (approximately 22-fold; Figure 1). This discrepancy may be attributed to several factors: (1) The fluorescence wavelength is shorter (490 nm) for C6 compared with that for TAMRA (532 nm) and is affected by the autofluorescence of the retina. (2) It is possible that the retinal delivery efficiency of SA-Lip is inferior to that of R8-Lip. (3) It is possible that the retinal delivery efficiency of thermoresponsive gels is inferior to that of liposomes alone. However, as will be discussed in greater detail later, the latter point is not supported by the results (Section 2.2.4). Regarding the thermoresponsive gel compositions, the SA-Lip-loaded SR-TG exhibited a significantly higher C6 fluorescence intensity compared with that in the SA-Lip-loaded SC-TG.

**Figure 4:**
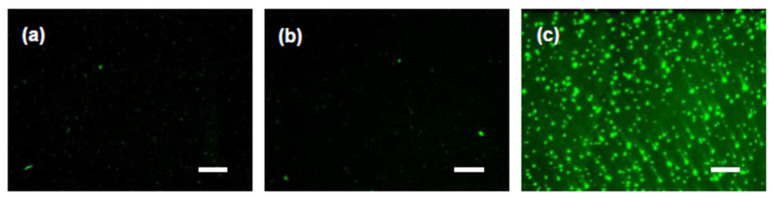
Fluorescence images of coumarin 6 (C6)-loaded liposomes in the thermoresponsive gel. SC, sodium citrate; SR, sorbitol; and TG, thermoresponsive gel. (**a**) Stearylamine-modified liposome (SA-Lip). (**b**) SA-Lip-loaded SR-TG. (**c**) SA-Lip-loaded SC-TG. Scale bar: 50 μm. The fluorescence derived from C6 was observed by fluorescence microscopy after heating each eye-drop formulation to 37 °C.

To further examine this discrepancy, fluorescence microscopy was used to assess the status of SA-Lip within the thermoresponsive gels (Figure 4). The liposomes in the SA-Lip-loaded SC-TG were aggregated into micro-sized particles (Figure 4c). As a salt, SC ionizes in aqueous solution and interacts with the hydration water on the liposomal surface, thereby reducing the hydration layer. This results in the aggregation of the liposomes because of the dehydrating effect of SC and the neutralization of the liposomal surface charge. During the evaluation of the particle properties (Section 2.2.1, Table 4), the thermoresponsive gel was sufficiently diluted, which may have reduced the liposomal aggregation derived from the SC. The SA-Lip-loaded SR-TG may initiate gelatinization after the eye drop, which may have resulted in the aggregation of SA-Lip and a subsequent reduction in retinal delivery efficiency. These results indicate that SR is a more effective gelatinization accelerator when positively charged liposomes are included, which potentially enhances the efficiency of retinal delivery. This finding is significant considering the limited reports on the particle properties of liposomes in thermoresponsive gels. A comprehensive and systematic examination is necessary to gain a deeper understanding of this phenomenon. Accordingly, SR-TG was used for subsequent studies.

#### 2.2.3. Liposome Retention Evaluation of the Thermoresponsive Gels

Centrifugation at 50,000 rpm (approximately 13,000× *g*) did not precipitate the liposomes. The presence of liposomes in the precipitate following centrifugation at 50,000 rpm indicated that they were retained within the thermoresponsive gel. Conversely, liposomes in the supernatant following centrifugation indicated that they were released from the thermoresponsive gel.

**Figure 5:**
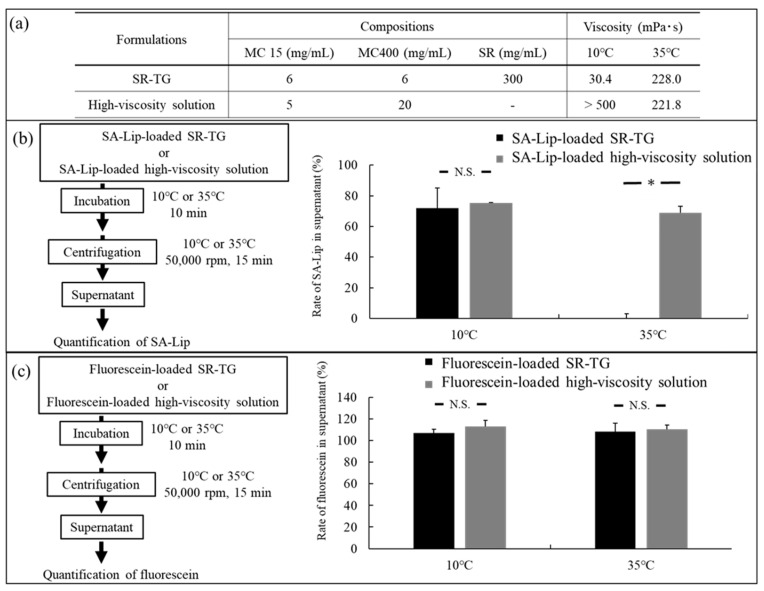
Liposome retention study of the thermoresponsive gels. MC, methylcellulose; SA-Lip, stearylamine-modified liposome; SR, sorbitol; and TG, thermoresponsive gel. (**a**) Composition and viscosity of the TG and high-viscosity solution. (**b**) Retention test of liposomes versus temperature. Rate of liposomes in the supernatant of the thermoresponsive gel or high-viscosity solution after centrifugation at various temperatures. (**c**) Retention of low-molecular-weight drugs (fluorescein) versus temperature. Rate of fluorescein in the supernatant of the thermoresponsive gel or high-viscosity solution after centrifugation at various temperatures. Each value represents the mean ± standard error of the mean (SEM) of three measurements. * *p* < 0.01 vs. SA-Lip-loaded high-viscosity solution (Aspin−Welch’s *t* test). N.S.: not significant.

Figure 5a shows the compositions and viscosities of the thermoresponsive gel and the high-viscosity solution. The SR-TG composition was modified to form a strong gel and ensure precipitation by centrifugation. The high-viscosity solution, which served as the control sample, was a high-viscosity MC solution devoid of gelatinization accelerators. The high-viscosity solution had the same viscosity as the SR-TG at 35 °C, without forming a gel network. Figure 5b shows the rate of SA-Lip in the supernatant following the centrifugation of the SA-Lip-loaded SR-TG and the SA-Lip-loaded high-viscosity solution. The rate of SA-Lip in the supernatant was similar in the high-viscosity solution at both 10 °C and 35 °C. In contrast, the rate of SA-Lip in the supernatant of SR-TG exhibited a notable decrease at 35 °C, suggesting that approximately 100% of the SA-Lip precipitated with the gelled SR-TG. To determine if the high retention of the thermoresponsive gels depends on particle size, fluorescein molecules smaller than SA-Lip were mixed with the SR-TG and the high-viscosity solution and centrifuged (Figure 5c). Fluorescein was present in the supernatant irrespective of the SR-TG temperature, confirming that it was not retained within the three-dimensional network structure of the thermoresponsive gel. The results indicate that the gelled thermoresponsive gel effectively retained liposomes with a particle size of approximately 100 nm. Thermoresponsive gels form a three-dimensional mesh structure at 35 °C, which is believed to be smaller than that of the liposomes [32]. We hypothesize that this high retention property contributes to the prolonged intraocular retention of liposomes, thereby reducing the frequency of eye-drop administration required.

#### 2.2.4. Effect of Liposome Retention of Thermoresponsive Gels on Mouse Retinal Delivery

**Figure 6:**
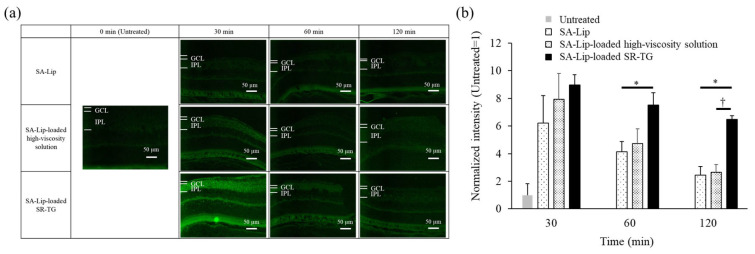
Mouse retinal delivery of coumarin 6 (C6) via single eye-drop administration of a stearylamine-modified liposome (SA-Lip). GCL, ganglion cell layer; IPL, inner plexiform layer; SR, sorbitol; and TG, thermoresponsive gel. (**a**) Time course of the epifluorescence microscopy images of the mouse retina. (**b**) Time course of the fluorescence intensity in the IPL. Each value represents the mean ± standard error of the mean (SEM) of four measurements. * *p* < 0.01 vs. SA-Lip, and † *p* < 0.05 vs. SA-Lip-loaded high-viscosity preparation (Aspin−Welch’s *t* test).

To evaluate the effect of liposome retention in thermoresponsive gels on mouse retinal delivery, the liposome suspension, high-viscosity solution, and thermoresponsive gel were administered via eye drops. Subsequently, mouse retinas were observed under a fluorescence microscope at 30, 60, and 120 min following administration (Figure 6).

The fluorescence intensity of the SA-Lip-loaded high-viscosity solution was consistently higher compared with that of SA-Lip alone. This result may be attributed to the increased viscosity, which prolongs the retention time on the ocular surface. Moreover, the fluorescence intensity of the SA-Lip-loaded SR-TG exhibited prolonged fluorescence. The fluorescence intensity was significantly higher for the SA-Lip-loaded SR-TG compared with that of the SA-Lip at 60 min and higher than that of the SA-Lip and SA-Lip-loaded high-viscosity solution at 120 min post-administration. These results indicate that the release of SA-Lip is regulated by the three-dimensional mesh structure formed by SR-TG. The consistently higher fluorescence intensity of the SA-Lip-loaded SR-TG compared with that of SA-Lip indicates that the loading of SA-Lip into the thermoresponsive gels does not result in reduced retinal delivery. These results support the concept that controlled release is an important factor in gelation, in conjunction with the viscosity of liposome formulations, to enhance the efficacy of liposome delivery to the retina.

#### 2.2.5. Retinal Edema Therapeutic Evaluation of Retinal-Vein Occlusion Model Mice After Liposome-Loaded Thermoresponsive Gel Eye-Drop Administration

Finally, we determined the therapeutic effect of liposome-loaded thermoresponsive gels on retinal edema. Thermoresponsive gels loaded with liposomes containing PnkRNA were administered to the eyes of RVO model mice. The R8-Lip from Section 2.1 was used to prepare the liposomes, whereas SR-TG was used to prepare the thermoresponsive gel. The particle properties of PnkRNA-loaded R8-Lip in SR-TG were comparable to those of SA-Lip (Table S2). We also demonstrated the delivery of C6-loaded R8-Lip on the retina (Figure S5).

**Figure 7:**
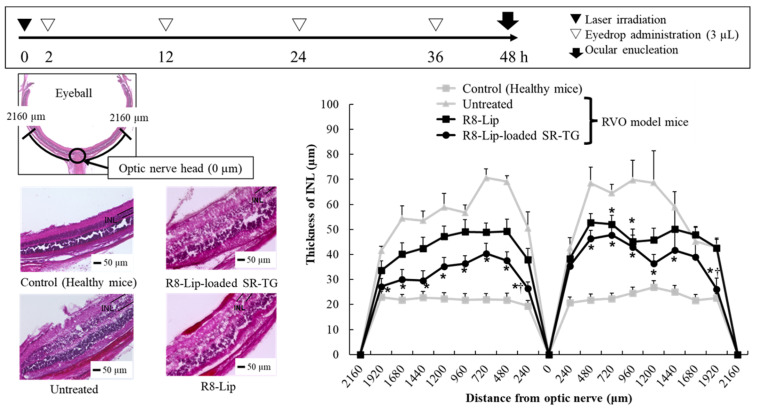
Thickness of the inner nuclear layer (INL) after specific intervals of eye-drop administration in retinal-vein occlusion (RVO) model mice. Lip, liposome; R8-Lip, stearoyl-octa-arginine (R8) modified liposome; SR, sorbitol; and TG, thermoresponsive gel. Each value represents the mean ± standard error of the mean (SEM) of eight measurements. * *p* < 0.05 vs. untreated, and † *p* < 0.05 vs. R8-Lip (one-way ANOVA followed by Tukey’s multiple comparison test). Note: the surface properties of the R8-Lip-loaded SR-TG are summarized in Table S2.

Figure 7 shows the evaluation scheme, retinal images, and INL thickness at 240 μm intervals centered on the optic nerve. The objective of using thermoresponsive gels was to diminish the frequency of eye-drop administration. Consequently, the number of eye-drop applications was reduced to four times over two days. The R8-Lip demonstrated a partial and significant suppression of INL thickening relative to that of the untreated group; however, the reduced number of eye drops resulted in an increase in INL thickness to twice that observed in the control group (healthy mice). In contrast, R8-Lip-loaded SR-TG demonstrated significant inhibition relative to that of the untreated group and showed partial, but statistically significant inhibition, compared with R8-Lip. The results showed that R8-Lip-loaded SR-TG eye drops effectively delivered PnkRNA to the test mouse retina and hold promise as an effective treatment for retinal edema in human RVO.

The current study provides proof of concept for the therapeutic efficacy of nucleic acid-loaded liposomes for RVO. This novel route of nucleic acid administration would eliminate the need for patients to visit a hospital and enable them to receive treatment at home. Moreover, the finding that nucleic acids can reach the retina from the ocular surface suggests that they may be effective at treating not only RVO, but a variety of retinal diseases, including age-related macular degeneration and diabetic retinopathy [33]. However, given that the liposomes are in suspension, there is a concern that transient blurred vision may occur following administration. Moreover, both R8-Lip and SA-Lip possess cationic surface potentials. Although the liposomes used in this study were suspended in HBSS–HEPES buffer at pH 7.4, making severe mucosal irritation unlikely, potential chronic adverse effects on vision and the risk of ocular tissue injury following instillation require further study. Because conventional RVO treatments, such as surgery and intravitreal injections, are invasive and require hospital visits, a thermoresponsive gel that reduces the frequency of eye-drop administration (four times over 2 days) offers a beneficial strategy to enhance patient safety and convenience. These nucleic acid-loaded liposomes offer a promising therapeutic strategy in the field of ophthalmic drug delivery.

## 3. Materials and Methods

### 3.1. Materials

The novel RNA oligonucleotides (PnkRNA, Bonac Co., Fukuoka, Japan) targeting mouse VEGF consisted of a 49-base single-stranded RNA and a self-annealing folding structure. The folded portion of the PnkRNA was replaced by a proline derivative (P). The sequence of the PnkRNA targeting mouse VEGF production was as follows: 5′-CGAUGAAGUCCUGGAGUGCGUCCPGGACGCACUCCAGGGCUUCAUCGUU-3′. The PnkRNA was dissolved in Hank’s balanced salt solution (HBSS, GIBCO) and 4-(2-hydroxyethyl)-1-piperazineethanesulfonic acid (HEPES, Nakalai Tesque Inc., Kyoto, Japan) buffer. The liposomes consisted of egg phosphatidylcholine (EPC, Nippon Oil and Fats Co., Ltd., Tokyo, Japan) and cholesterol (Sigma-Aldrich, St. Louis, MO, USA). Chloroform was purchased from Nakalai Tesque Inc. (Kyoto, Japan) for solvent extraction. The surface charge of the liposome was modified by stearoyl-octa-arginine (R8, BEX Co., Ltd., Tokyo, Japan) and stearylamine (SA, Tokyo Chemical Industry Co., Ltd., Tokyo, Japan). Two types of methylcellulose [methylcellulose #15 (MC15) and methylcellulose #400 (MC400)] were acquired from Nacalai Tesque as thermoresponsive polymers. Sorbitol (SR, Sorbit, Mitsubishi Corporation Life Sciences Ltd., Tokyo, Japan), tri-sodium citrate dihydrate (SC, Nacalai Tesque), and polyethylene glycol (PEG4000, Macrogol 4000, Maruishi Pharmaceutical Co., Ltd., Osaka, Japan) were purchased as gelatinization accelerators.

### 3.2. PnkRNA-Loaded Liposomal Eye Drops Inhibit Retinal Edema in the Retinal-Vein Occlusion Model Mice

#### 3.2.1. Preparation of PnkRNA-Loaded Liposomes by the Thin-Film Hydration Method

The thin-film hydration method was used to prepare PnkRNA-loaded liposomes. The compositional ratios of PnkRNA-loaded liposomes are listed in Table 1, and a comprehensive preparation scheme is illustrated in Figure S1. Briefly, all component solutes except PnkRNA were dissolved in chloroform and uniformly mixed in a round-bottom flask. The solvent was evaporated under reduced pressure, which yielded a thin lipid film. After completely removing the solvent, the film was hydrated with distilled water containing PnkRNA, thereby forming multilamellar vesicles. Subsequently, the multilamellar vesicles were adjusted to the desired particle size using an extruder (LipoFast™-Pneumatic; Avestin, Inc., Ottawa, ON, Canada) with a size-controlled polycarbonate membrane (membrane filter pore size: 100 nm, Whatman Japan KK, Tokyo, Japan). Finally, the liposome suspensions were dissolved in an HBSS–HEPES buffer (pH 7.4).

A Zetasizer Nano ZS (Malvern Instruments Ltd., Worcestershire, UK) was used to measure the particle properties, including particle size and zeta potential, of the PnkRNA-loaded liposomes. Before measurement, each liposome suspension was diluted 1000-fold with distilled water to prevent multiple scattering. To determine the loading efficiency of PnkRNA in the liposomes, fluorescence derived from a RiboGreen RNA Kit (ThermoFisher Scientific, Waltham, MA, USA) was measured. Subsequently, a five-fold dilution with Tris-EDTA buffer was performed, after which the liposome suspensions were centrifuged (4 °C, 75,000 rpm, 47 min) in a Himac CP80WX ultracentrifuge (Hitachi Ltd., Tokyo, Japan). The supernatant was collected and diluted 20-fold with Tris-EDTA buffer and mixed with an equal volume of RiboGreen. The amount of PnkRNA present in the supernatant was determined by calculating the fluorescence intensity of the RiboGreen fluorescence emission (510–570 nm) following 490 nm excitation, as detected by a GloMax-Multi Detection System (Promega Co., Madison, WI, USA). The percent loading efficiency of PnkRNA in the liposome suspension was calculated according to the following equation:

Loading efficiency (%) = 100 × (Preparation PnkRNA concentration − Supernatant PnkRNA concentration)/(Preparation PnkRNA concentration)

#### 3.2.2. Retinal Drug-Delivery Test of TAMRA-Labeled PnkRNA-Loaded Liposomal Eye Drops

The same methodology as described in Section 3.2.1 was used to prepare TAMRA-labeled PnkRNA-loaded liposomes. The composition is listed in Table 1. TAMRA-labeled PnkRNA was supplied by Bonac Corporation. An in vivo retinal drug-delivery test complied with the ARRIVE (Animal Research: Reporting of In Vivo Experiments) guidelines and was performed based on the Basic Guidelines for the Conduct of Animal Experiments of Gifu Pharmaceutical University (Approval number: 2019-040). Four-week-old male ddY mice were purchased from Japan SLC (Shizuoka, Japan) and housed in ventilated, temperature-controlled cages (25 °C) under a 12 h:12 h light/dark cycle with access to food and water ad libitum.

A 3 µL aliquot of the liposomal eye drops was administered to the mouse eyes using a micropipette six times at 5 min intervals. After 30 min, the mice were sacrificed, and their eyeballs were removed, and ocular tissue sections were immediately prepared. The eyeballs were rinsed with phosphate-buffered saline (PBS), pH 7.4, to remove any remaining PnkRNA that adhered to the surface. They were then immersed in a 4% paraformaldehyde phosphate buffer solution at 4 °C for 24 h. Subsequently, the eyeballs were transferred to a 20% sucrose phosphate buffer solution for 2 days at 4 °C. The eyeballs were embedded in Tissue-Tek^®^ O.C.T. Compound (Sakura Finetek Japan Co., Ltd., Tokyo, Japan), and a microtome (Cryostat CM1850, Wetzlar, Germany) was used to slice the retinal tissue into 10 µm thick sections with the optic nerve exposed. The distribution of the PnkRNA in the retina, located 500 µm from the optic nerve, was observed through a fluorescence microscope (BX51, Olympus Co., Tokyo, Japan) to detect the fluorescence derived from TAMRA. To assess the efficacy of PnkRNA delivery to the retina, ImageJ software (version 1.51j8, National Institutes of Health, Bethesda, MD, USA) was used to quantify the fluorescence intensity of TAMRA in the inner plexiform layer (IPL).

#### 3.2.3. Creation of the Retinal-Vein Occlusion Model Mice

To evaluate the therapeutic efficacy of PnkRNA-loaded liposomal eye drops, the RVO mouse model was established (approval number: 2020-084) [34]. Eight-week-old male ddY mice were anesthetized by an intraperitoneal injection of a mixture containing midazolam (4.0 mg/kg), medetomidine hydrochloride (0.3 mg/kg), and butorphanol tartrate (5.0 mg/kg). Rose Bengal (20 mg/kg, Fujifilm Wako Pure Chemical Co., Osaka, Japan), a photosensitizer, was administered into the tail vein, and 5 µL Midrin^®^P (0.5% tropicamide and 0.5% phenylephrine hydrochloride, Santen Pharmaceutical Co., Ltd., Osaka, Japan), a mydriatic agent, was administered to the eyes using a micropipette. A fundus imaging device (Micron4, Phoenix Research Laboratories, Inc., Pleasanton, CA, USA) equipped with a laser irradiation device (Meridian AG, Thun, Switzerland) was used to occlude the retinal vein in the right eye of the mouse by irradiating it three discs away from the optic disc with a laser (wavelength: 532 nm, spot size: 50 μm, irradiation time: 5 s, and laser power: 50 mW).

#### 3.2.4. Retinal Observation of the Retinal-Vein Occlusion Model Mice After PnkRNA-Loaded Liposome Eye-Drop Administration

The methods used to establish the RVO mouse model are described in Section 3.2.3. To demonstrate the efficacy of PnkRNA-loaded liposomal eye drops, the drops were administered to the RVO model mice. After laser irradiation, 3 µL of liposomal eye drops were administered to the right eye of the RVO model mice with a micropipette at predetermined times (2, 3, 6, 12, 18, 24, 30, and 36 h). HBSS solution was administered to other mice as a negative control (untreated group). The mice were sacrificed 48 h after laser irradiation, and their eyeballs were removed, washed with pH 7.4 PBS, and immersed in 4% paraformaldehyde PBS at 4 °C for 2 days. After fixation, the eyeballs were immersed sequentially in 5%, 10%, 15%, and 20% sucrose phosphate buffer for 2 h each, followed by 25% sucrose phosphate buffer overnight. The eyeballs were embedded in Tissue-Tek^®^ O.C.T. compound and sliced with a microtome. The retinal tissue sections were stained with hematoxylin and eosin, and the thickness of the inner nuclear layer (INL) was assessed by microscopy (BZ-9000, KEYENCE, Osaka, Japan). ImageJ software was used to quantify the INL thickness at 240 μm intervals centered on the optic nerve head.

### 3.3. Development of Liposome-Loaded Thermoresponsive Gels

#### 3.3.1. Preparation of Coumarin 6-Loaded Liposomes and Thermoresponsive Gels

The compositional ratios for the coumarin 6 (C6; MP Biomedical, Burlingame, CA, USA)-loaded liposomes are listed in Table 3, and the detailed preparation scheme is presented in Figure S2. The preparation scheme for the liposomes is described in Section 3.2.1. In this section, two surface modifiers, SA and R8, were used.

The process flow for the preparation of the thermoresponsive gels is shown in Figure S2. Briefly, MC15 and MC400 were dispersed in distilled water at 80 °C and stirred with gelatinization accelerators for 30 min. After stirring for 30 min at 50 °C, the MCs were dissolved by stirring for 60 min on ice. The thermoresponsive gels were produced by mixing equal numbers of the thermoresponsive gels and liposomes.

#### 3.3.2. Viscosity Test of the Thermoresponsive Gels

A viscosity meter (TV-10M, Toki Sangyo Co., Ltd., Tokyo, Japan) was used to measure the viscosity of the thermoresponsive gels. The gels were placed in a 200 mL jacketed beaker (Asahi Glassplant Inc., Kumamoto, Japan), which was connected to a digital thermos controller unit (UA-100; Tokyo Rikakikai Co., Ltd., Tokyo, Japan) to regulate the sample temperature between 10 °C and 40 °C. The thermoresponsive gels were measured by mixing equal volumes of gel and distilled water, as opposed to mixing a liposome suspension.

#### 3.3.3. Coumarin 6 Retinal Delivery Test of Liposome-Loaded Thermoresponsive Gels

A micropipette containing a 3 µL dose of liposome-loaded thermoresponsive gels was administered to the eyes of 4-week-old male ddY mice (Japan SLC) in a single dose. The ocular surface temperature was measured with a radiation thermometer (AD-5617, A&D Co., Ltd., Tokyo, Japan) before eye drop administration. The mice were sacrificed at 30, 60, or 120 min following treatment, and the eyeballs were removed. As detailed in Section 3.2.2, retinal tissue sections were prepared. The distribution of C6 in the retina, situated 500 µm from the optic nerve, was assessed under a fluorescence microscope.

#### 3.3.4. Images of the Liposomes in the Thermoresponsive Gels

A 5 µL aliquot of the liposome-loaded thermoresponsive gels was deposited onto a glass slide, and a cover slip was applied. The thermoresponsive gels were subjected to gelation in a water bath maintained at 37 °C. Subsequently, the samples were promptly examined for fluorescence using a fluorescence microscope before resolubilization.

#### 3.3.5. Retention Study of Liposomes from the Thermoresponsive Gels

The ability of the thermoresponsive gel to retain liposomes was determined by detecting the release of C6-loaded liposomes. The thermoresponsive gel and high-viscosity MC15 and MC400 solutions, prepared without a gelatinization accelerator, were mixed with equal quantities of loaded liposomes. The compositional ratios of the thermoresponsive gel and the high-viscosity solution are shown in Figure 5a. The thermoresponsive gel and high-viscosity solution were incubated in a water bath at 10 °C or 35 °C for 10 min and centrifuged at 50,000 rpm at 10 °C or 35 °C for 15 min. The percentage of C6-loaded liposomes in the supernatant was calculated using the GloMax-Multi Detection System, which measured the fluorescence emission of C6 at 510–570 nm with an excitation at 490 nm. The detailed calculation scheme is shown in Figure 5b,c. In addition, to determine the effect of liposomes, a thermoresponsive gel and a high-viscosity solution containing fluorescein sodium salt (Tokyo Chemical Industry Co.) were prepared, and their fluorescein sodium salt retention was evaluated.

#### 3.3.6. Retinal Observation of Retinal-Vein Occlusion Model Mice After Liposome-Loaded Thermoresponsive Gel Administration

The evaluation scheme was consistent with that presented in Section 3.2.4. In this section, R8-Lip-loaded SR-TG and R8-Lip were administered as eye drops to RVO model mice. The number of doses was set at four, and doses were administered at 2, 12, 24, and 36 h.

## 4. Conclusions

In this study, we developed a method for the retinal delivery of nucleic acid-loaded liposomes via eye-drop administration, which exhibited efficacy in an RVO mouse model. The results, which included nucleic acid detection by tissue section observation and INL-layer thickness in the mice, demonstrated that nucleic acid-loaded liposomes were delivered to the retina, taken up by retinal tissue, and resulted in therapeutic efficacy. The cationic modification of liposomes is important to ensure the loading rate and retinal delivery of nucleic acids. The use of thermoresponsive gels may reduce the number of liposome eye drops required for patients. It is important to note, however, that the constituents of the thermoresponsive gels may affect the particle properties of the liposomes.

## Figures and Tables

**Table 1:** Composition of PnkRNA-loaded liposomes.

Formulations	Surface Modifier	Concentration of EPC (mM)	Composition of the Liposomes (Molar Ratio)	Concentration of PnkRNA (µM)	Concentration of TAMRA-Labeled PnkRNA (µM)
Unmodified-Lip	―	10.2	EPC/Chol = 7/3	10	―
R8-Lip	R8	10.2	EPC/Chol/R8 = 7/3/0.175	10	―
Unmodified-Lip	―	10.2	EPC/Chol = 7/3	―	10
R8-Lip	R8	10.2	EPC/Chol/R8 = 7/3/0.175	―	10

Chol, cholesterol; EPC, egg phosphatidylcholine; Lip, liposome; and R8, stearoyl-octa-arginine.

**Table 2:** Particle properties of PnkRNA-loaded liposomes.

Formulations	Average Particle Size (nm)	Polydispersity	Zeta Potential (mV)	Loading Efficiency (%)
Unmodified-Lip	140.7	0.062	−12.0	18.8
R8-Lip	183.9	0.167	43.4	99.7
Unmodified-Lip	137.2	0.159	−1.0	18.8
R8-Lip	190.9	0.222	40.3	99.9

**Table 3:** Composition of coumarin 6 (C6)-loaded liposomes in the thermoresponsive gels.

Formulation	Composition								
	Surface Modifier	Concentration of EPC (mM)	Liposomal Composition (Molar Ratio)	Concentration of C6 (mg/mL)	MC 15 (mg/mL)	MC400 (mg/mL)	SR (mg/mL)	SC (mg/mL)	PEG4000 (mg/mL)
SA-Lip	SA	10.2	EPC/Chol/SA = 7/3/1	0.05	-	-	-	-	-
SA-Lip-loaded SR-TG	SA	10.2	EPC/Chol/SA = 7/3/1	0.05	20	-	20	-	-
SA-Lip-loaded SC-TG	SA	10.2	EPC/Chol/SA = 7/3/1	0.05	6	5	-	35	50

Chol, cholesterol; EPC, egg phosphatidylcholine; Lip, liposome; MC, methylcellulose; PEG, polyethylene glycol; SA, stearylamine; SC, sodium citrate; SR, sorbitol; and TG, thermoresponsive gel.

**Table 4:** Physicochemical properties of coumarin 6 (C6)-loaded liposomes in the thermoresponsive gels.

Formulation	Particle Properties			Viscosity (mPa·s)	
	Average Particle Size (nm)	Polydispersity	Zeta Potential (mV)	10 °C	35 °C
SA-Lip	141.6	0.074	65.0	<15	<15
SA-Lip-loaded SR-TG	265.8	0.343	5.2	30.8	46.6
SA-Lip-loaded SC-TG	353.4	0.460	−5.3	34.0	50.6

The particle properties of C6-loaded liposomes, which were diluted 1000-fold with distilled water before measurement at 15 °C to avoid the effects of multiple scattering and viscosity.

## Data Availability

Data will be made available on request.

## Supplementary Materials

The following supporting information can be downloaded at https://www.mdpi.com/article/10.3390/molecules30122622/s1, Figure S1: Process flow diagram of the preparation of PnkRNA-loaded liposomes; Figure S2: Process flow diagram of the preparation of the SA-Lip-loaded thermoresponsive gels; Figure S3: Relative VEGF-mRNA levels in retinal-vein occlusion (RVO) model mice after definite intervals of eye-drop administration; Figure S4: Mouse retinal delivery of coumarin 6 (C6) when stearoyl-octa-arginine–modified liposome (R8-Lip) is administered as a single eye drop to healthy mice and retinal-vein occlusion model mice; Figure S5: Mouse retinal delivery of coumarin 6 (C6) by single eye drop administration of stearoyl-octa-arginine–modified liposome (R8-Lip); Table S1: Compositions and viscosities of the thermoresponsive gels; Table S2: Particle properties of the R8-Lip-loaded SR-TG.

## Abbreviations

AAV	Adeno-associated virus
ANOVA	Analysis of variance
C6	Coumarin 6
COVID-19	Coronavirus disease 2019
DDS	Drug-delivery system
EPC	Egg phosphatidylcholine
GCL	Ganglion cell layer
HBSS	Hank’s balanced salt solution
HEPES	4-(2-hydroxyethyl)-1-piperazineethanesulfonic acid
INL	Inner nuclear layer
IPL	Inner plexiform layer
MC	Methylcellulose
PBS	Phosphate-buffered saline
PEG	Polyethylene glycol
R8	Stearoyl-octa-arginine
RVO	Retinal-vein occlusion
SA	Stearylamine
SC	Tri-sodium citrate dihydrate
SR	Sorbitol
TGF-β1	Transforming growth factor-β1
VEGF	Vascular endothelial growth factor
