# Supplementary material for: Topical Application of RNAi Therapy Using Surface-Modified Liposomes for Treating Retinal-Vein Occlusion
Source: Molecules. 2025 Jun 17;30(12):2622. doi: 10.3390/molecules30122622 (PMC12196029; doi:10.3390/molecules30122622)
Supplement: Supplementary file 1 [file molecules-30-02622-s001.zip › molecules-3650397-supplementary.pdf]

SUPPLEMENTARY INFORMATION

Topical Application of RNAi Therapy Using Surface-Modified Liposomes for Treating Retinal-Vein Occlusion

Taishi Shiratori [1,†], Takaaki Ito [1,†], Anri Nishinaka [2], Ryosuke Matsumiya [1], Eriko Yamazoe [1], Hirofumi Takeuchi [1,3], Hideaki Hara [2] and Kohei Tahara [1,*]

1 Laboratory of Pharmaceutical Engineering, Gifu Pharmaceutical University, 1-25-4 Daigaku-Nishi, Gifu 501-1196, Japan; ito-ta@gifu-pu.ac.jp (T.I.); lucky.ryosuke.0414@gmail.com (R.M.); yamazoe-e@gifu-pu.ac.jp (E.Y.); takeuchi@gifu-pu.ac.jp (H.T.)
2 Laboratory of Molecular Pharmacology, Gifu Pharmaceutical University, 1-25-4 Daigaku-Nishi, Gifu 501-1196, Japan; hidehara@gifu-pu.ac.jp (H.H.)
3 Laboratory of Advanced Pharmaceutical Process Engineering, Gifu Pharmaceutical University, 1-25-4 Daigaku-Nishi, Gifu 501-1196, Japan
* Correspondence: tahara@gifu-pu.ac.jp; Tel.: +81-58-230-8115
† These authors contributed equally to this study.

Supplementary methods

VEGF-mRNA levels in retinal-vein occlusion model mice after PnkRNA-loaded liposome eye-drops administration

The methods used to establish the RVO-model mice and administer the eye drops were the same as those described in Sections 2.2.3 and 2.2.4. After 48 h of laser irradiation, the mice were sacrificed and their eyeballs were removed. The retinal tissue was carefully isolated from the eyeballs and quickly frozen in liquid nitrogen. The frozen retinal tissue was then defrosted on ice and homogenized in a homogenizer (Power Masher II, Nippi Co., Tokyo, Japan). An RNA extraction kit (NucleoSpin®, Takara Bio Inc., Shiga, Japan) was used to extract the total RNA from the retina, and a UV–Visible spectrophotometer (NanoVue Plus, Biochrom, MA, USA) was used to measure the absorbance at 260 nm to enable calculation of the total RNA concentration in the samples. After quantification of the total RNA concentration, the RNA concentration was standardized between samples by use of RNase-free water (Takara Bio Inc.).

A thermal cycler (CFX96, Bio-Rad, CA, USA) and reverse transcriptase (PrimeScript™ RT reagent Kit, Takara Bio Inc.) according to the protocol were used to synthesize cDNA from the total RNA of the retinal tissue. VEGF-mRNA levels were normalized to the amount of the housekeeping gene encoding mouse glyceraldehyde-3-phosphate dehydrogenase (GAPDH) by the ΔΔCT method of reverse transcription-polymerase chain reaction (RT-PCR). The PCR reaction reagent consisted of TB Green Premix Ex Taq II (Takara Bio Inc.), and primers were added to the cDNA samples. The threshold cycle (Ct value) of PCR amplification of the mouse VEGF and mouse GAPDH was determined using a thermal

cycler (CFX96). The primer sequences for mouse VEGF and mouse GAPDH were as follows:

Mouse VEGF primer: Forward 5′ –3′ : 5′ -ACATTGGCTCACTTCCAGAAACAC-3′ ; Reverse 5′ –3′ : 5′ -GGTTGGAACCGGCATCTTTATC-3′ .

Mouse GAPDH primer: Forward 5′ –3′ : 5′ -CACATTGGGGGTAGGAACAC-3′ ; Reverse 5′ –3′ : 5′ -AACTTTGGCATTGTGGAAGG-3′ .

Supplementary Figures and Tables

Table S1. Compositions and viscosities of the thermoresponsive gels.

Formulations	Compositions					Viscosity (mPa・s)			
	MC15 (mg/mL)	MC400 (mg/mL)	SR (mg/mL)	SC (mg/mL)	PEG4000 (mg/mL)	10℃	25℃	35℃	40℃
SR-TG 1[α]	20	-	200	-	-	30.8	18.9	46.6	463.2
SR-TG 2	10	10	200	-	-	217.0	111.0	111.0	91,500.0
SR-TG 3	-	20	200	-	-	1434.0	3,800.0	142,400.0	No data
SR-TG 4	10	10	100	-	-	155.0	78.9	57.1	50.2
SR-TG 5	10	10	150	-	-	No data	109.3	573.0	3,488.0
SR-TG 6	10	10	300	-	-	164.0	91.2	530.0	608.0
SR-TG 7*	6	6	300	-	-	30.4	No data	228.0	No data
SC-TG 1	7	7	-	35	20	No data	37.9	400.7	1934.0
SC-TG 2	6	6	-	35	50	47.0	No data	294.2	754.0
SC-TG 3	6	6	-	35	20	39.3	No data	36.4	No data
SC-TG 4	6	5	-	35	20	29.9	78.9	14.4	23.2
SC-TG 5[α]	6	5	-	35	50	34.0	18.9	50.6	420.4

MC, methylcellulose; PEG, polyethylene glycol; SC, sodium citrate; SR, sorbitol; TG, thermoresponsive gel

Comprehensive studies of the compositions were evaluated on thermoresponsive gels without liposomes. α: SR-TG 1 and SC-TG5 were used in this study as SA-Lip-loaded SR-TG and SA-Lip-loaded SC-TG.

Table S2. Particle properties of the R8-Lip-loaded SR-TG.

Formulations	Average particle size (nm)	Polydispersity	Zeta potential (mV)
PnkRNA loaded R8-Lip SR-TG	158.8	0.177	13.2
C6 loaded R8-Lip SR-TG	125.4	0.178	12.4

C6, coumarin 6; Lip, liposome; R8, stearoyl-octa-arginine; SR, sorbitol; TG, thermoresponsive gel

The C6-loaded R8-Lip SR-TG was used in Fig. S5.

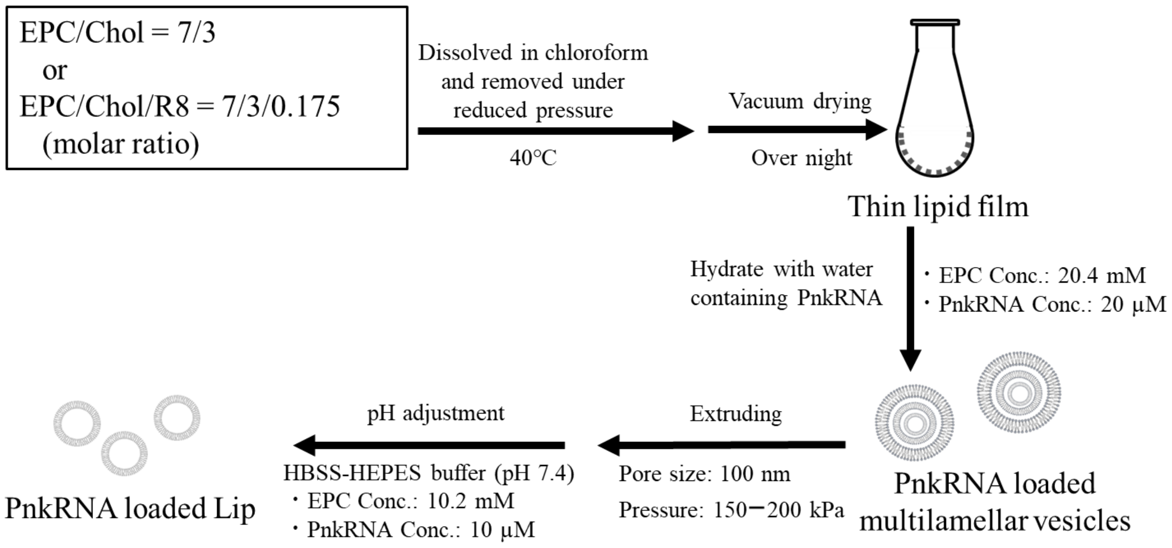

Figure S1. Process flow diagram of the preparation of PnkRNA-loaded liposomes.

Chol, cholesterol; EPC, egg phosphatidylcholine; HBSS, Hank's balanced salt solution; HEPES, 4-(2-hydroxyethyl)piperazine-1-ethane-sulfonic acid; Lip, liposome; R8, stearoyl-octa-arginine.

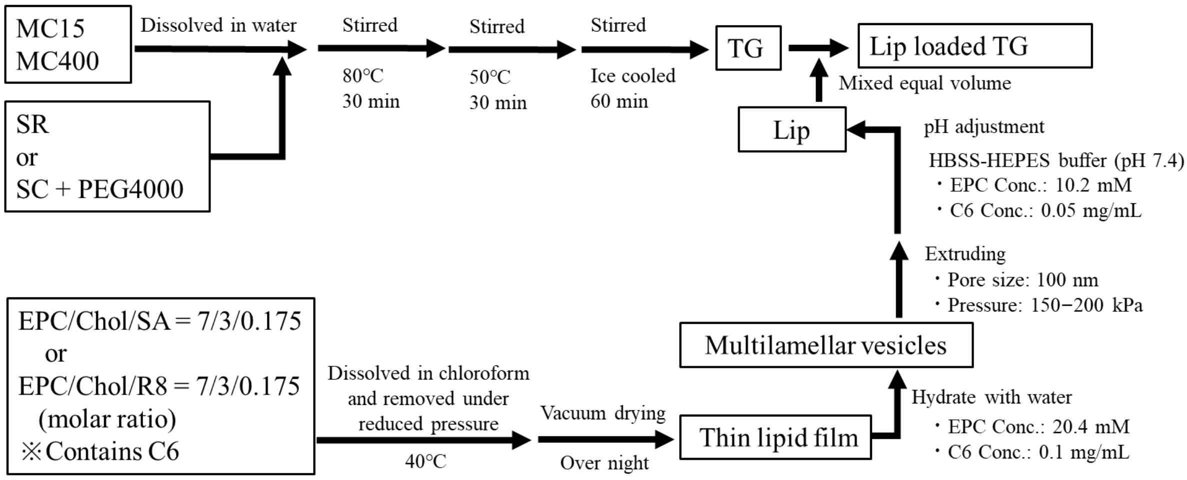

Figure S2. Process flow diagram of the preparation of the SA-Lip-loaded thermoresponsive gels.

C6, coumarin 6; Chol, cholesterol; EPC, egg phosphatidylcholine; HBSS, Hank's balanced salt solution; HEPES, 4-(2-hydroxyethyl)piperazine-1-ethane-sulfonic acid; Lip, liposome; MC, methylcellulose; PEG, polyethylene glycol; R8, stearoyl-octa-arginine; SA, stearylamine; SC, sodium citrate; SR, sorbitol; TG, thermoresponsive gel

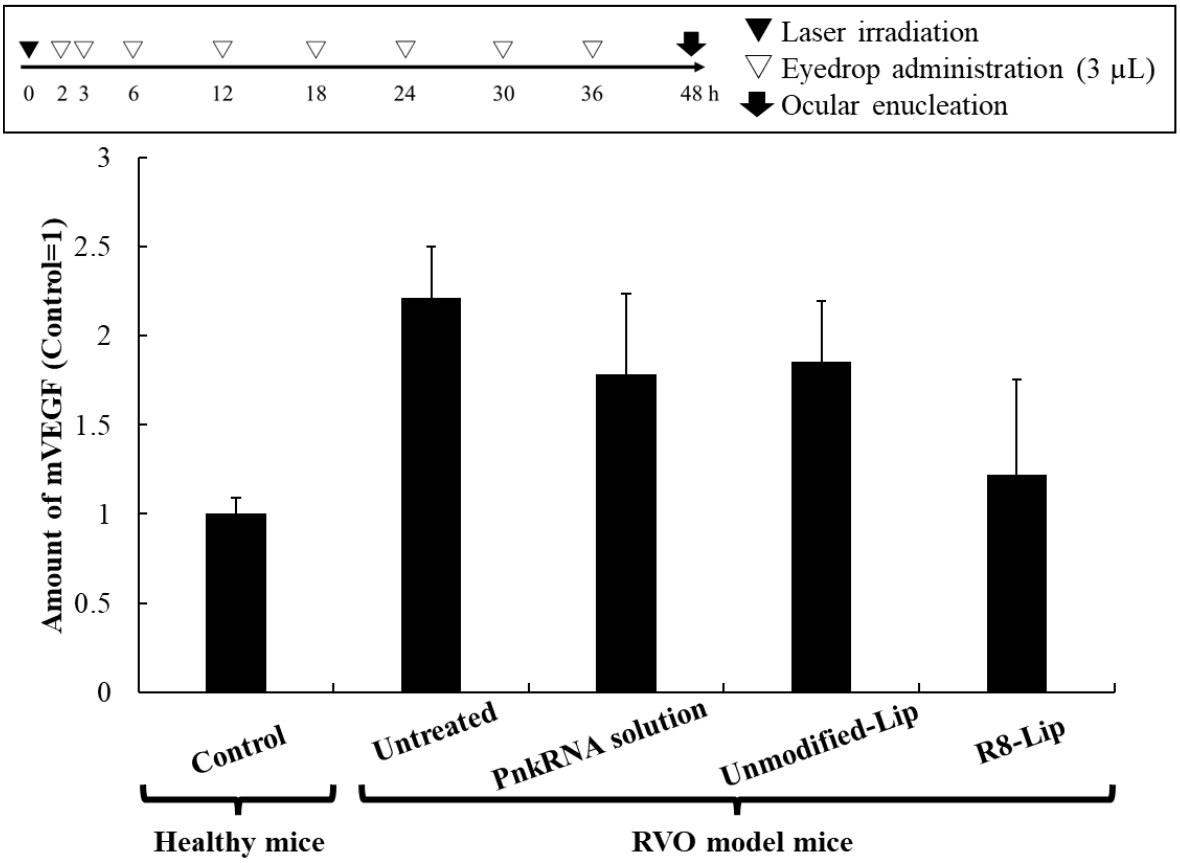

Figure S3. Relative VEGF-mRNA levels in retinal-vein occlusion (RVO) model mice after definite intervals of eye-drop administration.

Lip, liposome; R8-Lip, stearoyl-octa-arginine (R8) modified liposome. The VEGF-mRNA level was analyzed by applying the ΔΔCt method (n = 4, mean ± standard error of the mean [SEM]). Compared with the Untreated group, there were no significant differences (Aspin−Welch's *t* test).

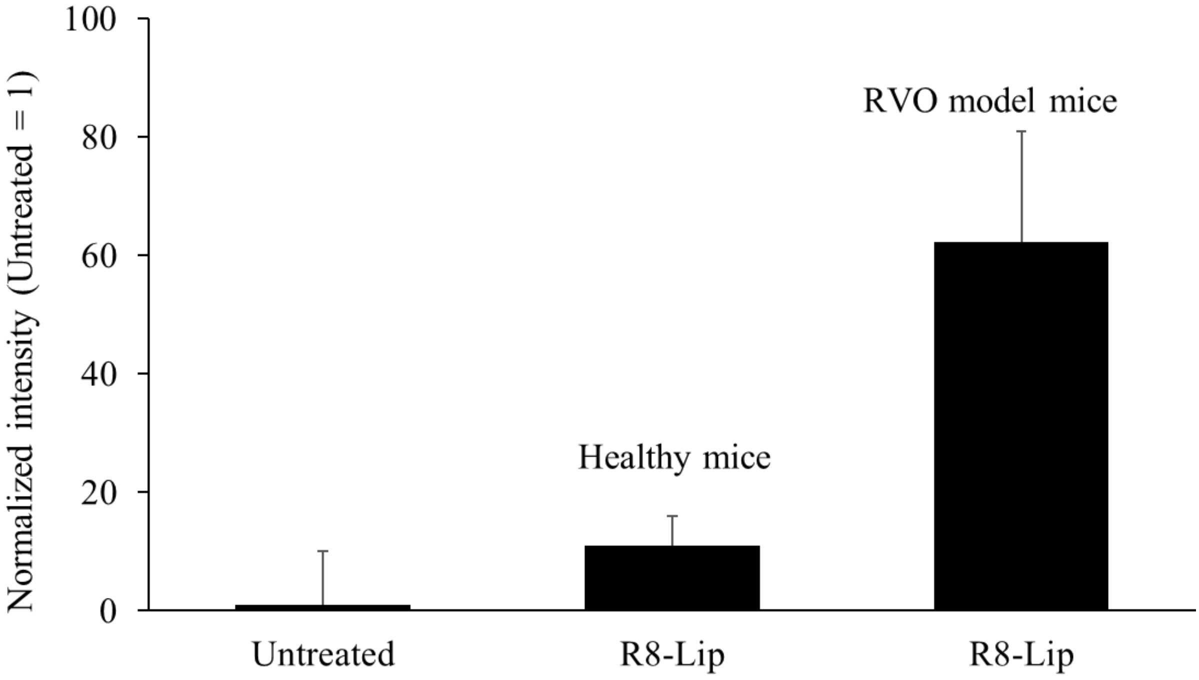

Figure S4. Mouse retinal delivery of coumarin 6 (C6) when stearoyl-octa-arginine–modified liposome (R8-Lip) is administered as a single eye drop to healthy mice and retinal-vein occlusion model mice.

Time course of the accumulated fluorescence intensity in the inner plexiform layer (IPL).

Each value represents the mean ± standard error of the mean (SEM) of the four measurements.

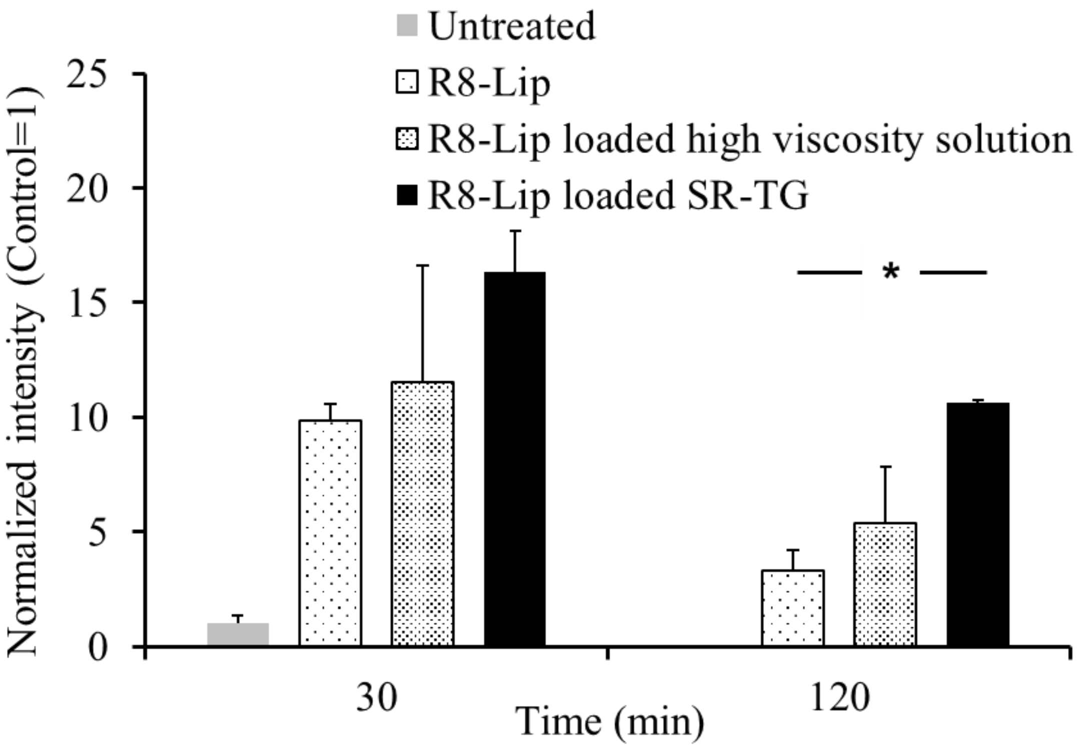

Figure S5. Mouse retinal delivery of coumarin 6 (C6) by single eye drop administration of stearoyl-octa-arginine–modified liposome (R8-Lip).

Time course of the accumulated fluorescence intensity in the inner plexiform layer (IPL).

Each value represents the mean ± standard error of the mean (SEM) of four measurements.

**P* < 0.01 vs. R8-Lip (Aspin–Welch's *t* test).
